# Acute Necrotizing Esophagitis Followed by Duodenal Necrosis

**DOI:** 10.4021/gr361w

**Published:** 2011-11-20

**Authors:** Piedad Magdalena del Hierro

**Affiliations:** a Ecuadorian Society of Gastroenterology. Av. Gil Ramirez Davalos 1-33. Cuenca-Ecuador. Email: magdalenadelhierro@yahoo.com

**Keywords:** Esophagitis, Necrosis, Black esophagus, Gastrointestinal bleeding, Duodenal necrosis

## Abstract

Acute Necrotizing Esophagitis is an uncommon pathology, characterized by endoscopic finding of diffuse black coloration in esophageal mucosa and histological presence of necrosis in patients with upper gastrointestinal bleeding. The first case of acute necrotizing esophagitis followed by duodenal necrosis, in 81 years old woman with a positive history of Type 2 Diabetes Mellitus, Hypertension, and usual intake of Nonsteroidal Anti-inflammatory drugs, is reported. Although its etiology remains unknown, the duodenal necrosis suggests that ischemia could be the main cause given that the branches off the celiac axis provide common blood supply to the distal esophageal and duodenal tissue. The massive gastroesophagic reflux and NSAID intake could be involved.

## Introduction

Acute Necrotizing Esophagitis (ANE), or “Black esophagus”, is an uncommon pathology, characterized by endoscopic finding of diffuse black coloration in esophageal mucosa and histological presence of necrosis. Clinically debuts as upper gastrointestinal bleeding (UGB).

Although, Black Esophagus was found previously in necropsies [1], the first endoscopic description was made in 1990 [2], since then, many authors have reported its appearance associated with different diseases. There are no reports that link ANE with duodenal necrosis.

## Case Report

An 81 years old woman was transferred to the hospital for assessment after presenting hematemesis and epigastralgia. The patient had a positive history of Type 2 Diabetes Mellitus (DM), Hypertension, and usual intake of Nonsteroidal Anti-inflammatory drugs (NSAID) for joint pain. Physical examination revealed painful epigastria on deep palpation, and the presence of black stools on rectal ampoule.

Laboratory values included: White blood cells count 22 300/µL (4 000-10 000/µL) with 87.7% neutrophills (40 - 70%), Hemoglobin 11.9 g/dL (12 - 15 g/dL), Glucose 74.1 mg/dL (70 - 110 mg/dL), Urea 148 mg/dL (20 - 40 mg/dL), Creatinine: 2.76 mg/dL (0.50 - 1.20 mg/dL), Albumin 1.8 g/dL (3.4 - 5 g/dL).

**Figure 1:**
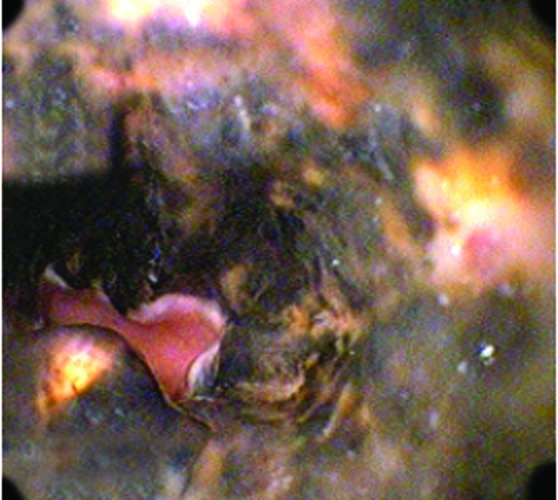
Endoscopic Image demonstrating diffuse esophageal necrosis.

EGD showed the esophagus with circumferential black coloration extending from 23 cm to the gastro-esophageal junction (Fig 1), small erosions covered with necrotic tissue in gastric body and congestive duodenal mucosa. Treatment with Omeprazol and Sucralfato was started.

**Figure 2:**
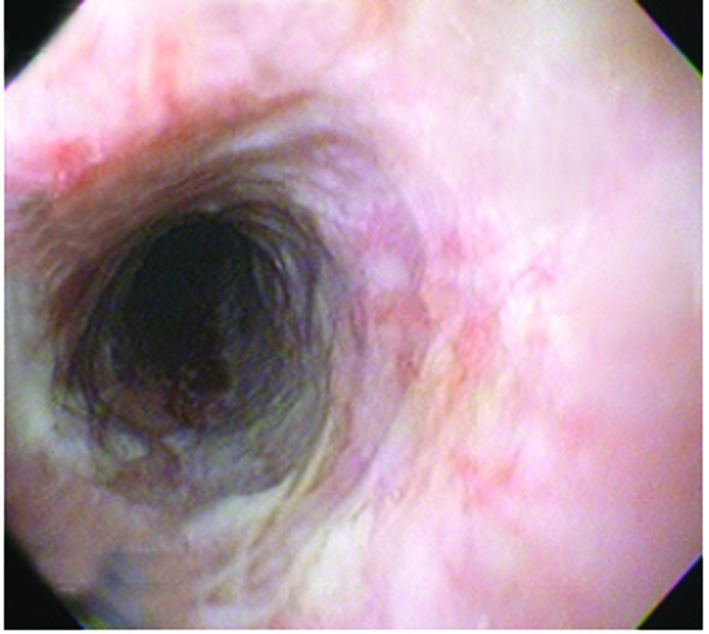
Endoscopic Image eleven days after admission shows recovery of the necrotic esophageal mucosa.

On the fifth day EGD showed improvement of esophageal injuries, and cotton infiltrate, biopsy samples were taken. Histological report revealed wide necrotic areas, spores and hyphae. Mycological report revealed Candida Mycelium. Fluconazol was added to previous treatment. The patient evolved well and was discharged six days later with no endoscopic evidence of necrosis (Fig 2).

**Figure 3:**
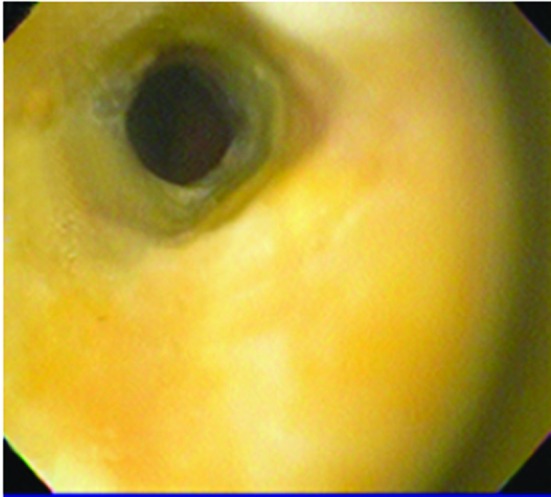
Endoscopic Image demonstrating yellow discoloration of esophageal mucosa four months after presenting acute necrotizing esophagitis.

**Figure 4:**
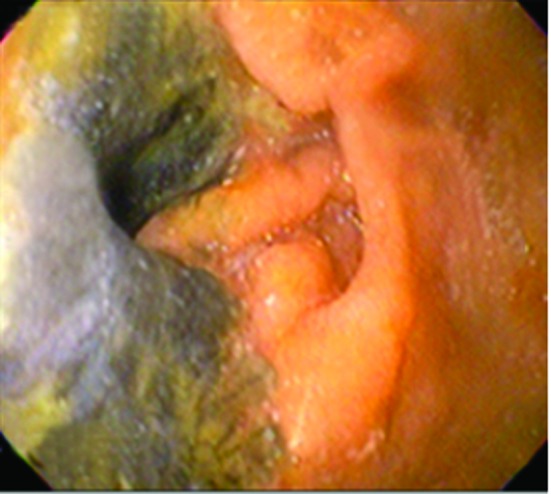
Endoscopic Image demonstrating duodenal necrosis in the same patient four month later.

Four months later, the patient came back with jaundice and right upper quadrant pain. EGD showed esophagus mucosa with yellow discoloration (Fig 3), and necrotic areas on the second portion of duodenum (Fig 4). Histology report informed dense inflammatory infiltrate and duodenal mucosa necrosis. The cause of jaundice couldn’t be determinated, and three weeks later the patient died.

## Discussion

The black esophagus is a rare entity, which incidence has not been defined [3, 4], some studies show that it may be between 0.01% [5, 6] and 0.28 % [4]. A study performed among Japanese patients with UGB found an incidence of 6% [7].

The appearance of ANE has been associated with hypothermia [1], Peripheral arterial disease [8], Alcohol abuse [9], cirrhosis [10], Diabetic ketoacidosis [7], cholangiocarcinoma [11], malnutrition, nephrotic syndrome [12], DM, hypertension, and NSAID intake [7].

The etiology of ANE remains unknown, some authors believe that ischemia is the main cause [2-4, 13], but massive gastroesophagic reflux [14] and NSAID intake could also be involved [7], It seems possible that all of these factor had contributed to development of ANE in this patient, this supports the idea that etiology could be multifactorial [15].

Duodenal necrosis after the appearance of ANE suggests that they share the same etiology. Endothelial damage caused by DM, hypertension, renal failure could have contributed to an ischemic event becomes the cause of necrosis. Vascular compromise of branches off the celiac axis, that provides common blood supply to the distal esophageal and duodenal tissue can also explain duodenal bulb ulcers, erosions, inflammation, edema of duodenum commonly seen in association with ANE [15]. Vascular insufficiency decreases the buffer ability of the mucosal and makes it vulnerable to damage [16]. NSAID intake can produce injury in all the gastrointestinal tract included duodenum and esophagus. Diabetic gastroparesia could cause massive gastro-esophagical reflux which could damage an esophageal mucosa already week by ischemia.

There are no reports of yellow coloration of esophagus, a case of ANE with yellow exudates was reported in a patient with cholestasis [17]. Maybe this could be a rare clinical feature of jaundice in patients with susceptible esophageal mucosa or impairment in esophageal vasculature.
